# Safety Surveillance of Pneumococcal Vaccine Using Three Algorithms: Disproportionality Methods, Empirical Bayes Geometric Mean, and Tree-Based Scan Statistic

**DOI:** 10.3390/vaccines8020242

**Published:** 2020-05-22

**Authors:** Hyesung Lee, Ju Hwan Kim, Young June Choe, Ju-Young Shin

**Affiliations:** 1 School of Pharmacy, Sungkyunkwan University, Suwon 16419, Korea; gul2@skku.edu (H.L.); napa928@hotmail.com (J.H.K.); 2 College of Medicine, Hallym University, Chuncheon 24252, Korea; ychoe@hallym.ac.kr; 3 Samsung Advanced Institute for Health Sciences and Technology (SAIHST), Sungkyunkwan University, Seoul 06355, Korea

**Keywords:** tree-based scan statistics, empirical Bayes geometric mean, quantitative signal detection, pneumococcal vaccine

## Abstract

*Introduction:* Diverse algorithms for signal detection exist. However, inconsistent results are often encountered among the algorithms due to different levels of specificity used in defining the adverse events (AEs) and signal threshold. We aimed to explore potential safety signals for two pneumococcal vaccines in a spontaneous reporting database and compare the results and performances among the algorithms. *Methods:* Safety surveillance was conducted using the Korea national spontaneous reporting database from 1988 to 2017. Safety signals for pneumococcal vaccine and its subtypes were detected using the following the algorithms: disproportionality methods comprising of proportional reporting ratio (PRR), reporting odds ratio (ROR), and information component (IC); empirical Bayes geometric mean (EBGM); and tree-based scan statistics (TSS). Moreover, the performances of these algorithms were measured by comparing detected signals with the known AEs or pneumococcal vaccines (reference standard). *Results:* Among 10,380 vaccine-related AEs, 1135 reports and 101 AE terms were reported following pneumococcal vaccine. IC generated the most safety signals for pneumococcal vaccine (40/101), followed by PRR and ROR (19/101 each), TSS (15/101), and EBGM (1/101). Similar results were observed for its subtypes. Cellulitis was the only AE detected by all algorithms for pneumococcal vaccine. TSS showed the best balance in the performance: the highest in accuracy, negative predictive value, and area under the curve (70.3%, 67.4%, and 64.2%). *Conclusion:* Discrepancy in the number of detected signals was observed between algorithms. EBGM and TSS calibrated noise better than disproportionality methods, and TSS showed balanced performance. Nonetheless, these results should be interpreted with caution due to a lack of a gold standard for signal detection.

## 1. Introduction

Algorithms for signal detection have been developed and successfully implemented in medical databases for post-marketing drug safety surveillance [1]. These algorithms have demonstrated varying degrees of sensitivity and specificity in multiple medical databases, leaving the drug safety monitoring authorities to choose from the algorithms that are practical and validated in their regional databases. Mostly, the disproportionality methods comprising of proportional reporting ratio (PRR), reporting odds ratio (ROR), and information component (IC) are used, and the United States Food and Drug Administration (FDA) uses the empirical Bayes geometric mean (EBGM) [2,3]. Although these algorithms have been previously validated, inconsistent signal detection results are often encountered within and between the algorithms due to different levels of specificity in defining adverse events (AEs) and signal score threshold [4,5].

A study by Kulldorff et al. introduced a new signal detection algorithm, the tree-based scan statistic, which, unlike other algorithms based on rate ratios or Bayesian shrinkage estimator, is based on a log-likelihood ratio-based approach and adjust for multiple-testing. [6,7]. Moreover, this quantitative method is suitable for handling hierarchy structured variables such as AE terminology and anatomical therapeutic chemical (ATC) classification system [8]. As signal detection through this method is fundamentally based on a pre-defined tree structure constructed with the variables of interest grouped together at different specificity levels, it can both evaluate a variable alone and a group of related variables simultaneously [9]. 

*Streptococcus pneumoniae* is a major pathogen associated with substantial morbidity and mortality worldwide [10]. This pathogen can lead to invasive pneumonia, which, in turn, leads to high morbidity and mortality, especially in children and the elderly [11]; it was the fourth cause of mortality in Korea in 2012 [12]. The regulatory agency of South Korea introduced pneumococcal vaccines into the National Immunization Program (NIP) for the elderly (23-valent pneumococcal polysaccharide vaccines; PPSVs) and for children (10- and 13-valent pneumococcal conjugate vaccines; PCVs) in 2013 and 2014, respectively [13]. Ever since, not only the immunization rate for PPSV/PCV [14] but also the volume of AE reports increased substantially upon their introduction in the NIP [15]. 

Given the limited application of data mining for safety signal detection and post-marketing safety data for pneumococcal vaccines in the domestic spontaneous reporting system, we aimed to identify potential safety signals of pneumococcal vaccine using the disproportionality methods, EBGM, and tree-based scan statistic. Additionally, the performance of each algorithm was evaluated using a reference standard to demonstrate the applicability of these algorithms for vaccine safety surveillance. 

## 2. Materials and Methods

### 2.1. Data Sources

We used data from the Korea Institute of Drug Safety & Risk Management-Korea Adverse Event Reporting System Database (KIDS-KD). The Korean government introduced the spontaneous reporting system for AEs in 1988 [16] and established the Korea Adverse Event Reporting System (KAERS) in 2012 to facilitate the reporting and management of AEs after administration of medications, therapeutic biologic products, and vaccines [17]. The KIDS-KD is collected through the KAERS, and contains the information on demographics, AEs, drug, reporter, and a causality assessment. All drugs and AEs were coded according to the ATC code and the World Health Organization-Adverse Reaction Terminology (WHO-ART), respectively. The WHO-ART is constructed as a tree structure [18], and we used the level of preferred terms (PTs) that represent the principal terminology used for documentation. 

### 2.2. Study Vaccine

We included all NIP and non-NIP vaccines listed in routine vaccination schedule in Korea for this study. The study vaccine was pneumococcal vaccine approved in Korea, and its subtype, 23-valent PPSV (Prodiax-23^®^/PNEUMOVAX 23^®^) and 10- and 13-valent PCV (Synflorix^®^ pre-filled syringe and Prevenar 13^®^, respectively). The other vaccines used as a comparator for signal detection are listed in the Table S1. 

### 2.3. Selection of AE Reports

From the entire AE reports in KIDS-KD from 1988 to 2017, we used the initial reports that were reported following vaccination. In other words, we excluded AE reports involving pharmaceuticals other than vaccine or with unspecified vaccine product and follow-up report for initially reported AEs. We also excluded the reports without causality assessment or recorded as “unlikely”, “unclassified”, or “unassessable” according to the WHO causality assessment criteria. Lastly, we excluded the reports that listed both PPSV and PCV administered at the same date as these can complicate the interpretation of the signals. 

### 2.4. Statistical Analysis

#### 2.4.1. Descriptive Analysis 

We provided the frequency and proportion of basic characteristics (sex, age group, the type of reports, the report source by professions, and report source by affiliation) to compare their distributions between pneumococcal vaccines and all other vaccines. In addition, we described the list of AEs according to the System Organ Class (SOC) for pneumococcal vaccine, including both subtypes, and all other vaccines. Moreover, we conducted subgroup analysis to identify the difference of AE distribution according to three age groups: (1) 19 years old or younger; (2) 19–64 years old (3) 65 years old or older. 

#### 2.4.2. Algorithms for Signal Detection

Signal is information about a potential relationship between a drug and AE [19]. Although a signal does not indicate a causal relationship, it provides preliminary evidence for establishing a safety profile. In order to detect the signals for the pneumococcal vaccine and its subtypes, we used the tree-based scan statistic and two widely used methods, disproportionality methods, and EBGM. 

##### Disproportionality Method

In this study, we generated signal scores of all AE-pairs for the pneumococcal vaccine using the PRR, ROR and IC, which are the algorithms employed by Korea Institute of Drug Safety & Risk Management for routine drug safety surveillance [20]. Thresholds for each measurement were established as follow: (a) PRR: ≥2; (b) ROR: ≥2 (c) IC: ≥0. Additionally, chi-square statistics and frequency of AEs were considered with the results of disproportionality methods, simultaneously: (a) chi-square: ≥2; (b) frequency: ≥3.

##### Empirical Bayes Geometric Mean (EBGM)

The EBGM was introduced to detect signals for large counts in contingency tables by using observed and expected counts for each AE-pair [21]. It reduces false positive signaling by shrinking large measurements with small counts. We defined the threshold as the lower bound of the 90% confidence interval of the EBGM greater or equal to 2, which is the previously validated threshold employed by the FDA for routine drug safety surveillance [22]. 

##### Tree-based Scan Statistic 

The tree-based scan statistic is based on log-likelihood ratio statistics and multiple testing and is suitable for analyzing a hierarchical structure variable [23]. We calculated the expected value by tabulating a two by two table for each AE-pair under the null hypothesis. The threshold of the *p*-value to identify a safety signal was defined as 0.05. Using the unconditional Poisson model, AEs were defined as signals when their *p*-value was lower than 0.05, and *p*-values were generated with Monte Carlo simulation. 

#### 2.4.3. Performance Evaluation 

We conducted a performance evaluation to identify which algorithm was more suitable for detecting safety signals for the pneumococcal vaccines. We established a reference standard by reviewing the adverse reaction section of the FDA-approved package inserts and the labeling information approved by the Ministry of Food and Drug Safety of South Korea. AEs listed on the package inserts [24] or in the labeling information [25] were used to constuct the reference standard. We then constructed a confusion matrix for each algorithm, comparing the detected signals with the reference standard to calculate: (a) accuracy; (b) sensitivity; (c) specificity; (d) positive predicted value (PPV); (e) negative predicted value (NPV); (f) area under the curve (AUC). Detailed formula used for the performance evaluation is explained in the Figure S1 [26,27]. 

To account for effect modification by age, we also conducted subgroup analyses for the following subgroups: (1) 19 years old or younger; (2) 19–64 years old (3) 65 years old or older. All statistical analyses were performed using SAS 9.4 for Windows (SAS Institute, Inc., Cary, NC, USA), R Statistical Software version 3.5.1 (R Foundation for Statistical Computing, Vienna, Austria), and TreeScan^®^ software version 1.4. The study protocol was approved by the Sungkyunkwan University Institutional Review Board (No. 2019-09-005). 

## 3. Results

### 3.1. General Characteristics

**Figure 1:**
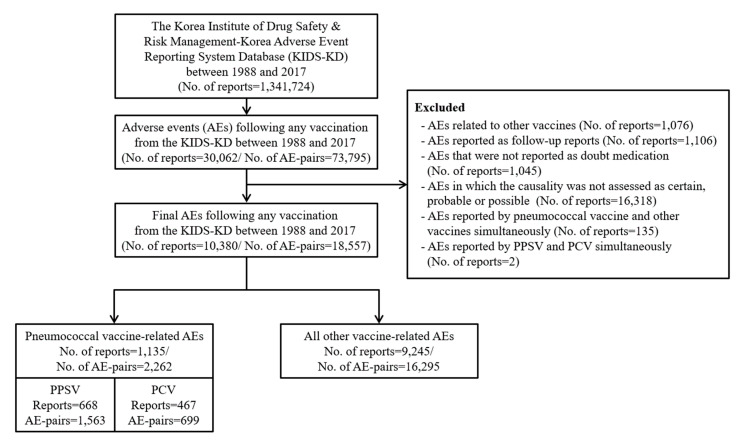
Flowchart of selection for adverse event reports. Abbreviations: PPSV, pneumococcal polysaccharide vaccine; PCV, pneumococcal conjugate vaccine.

From a total of 1,341,724 reports in the KAERS, we identified 30,062 (2.2%) reports involving vaccination (Figure 1). After applying our study exclusion criteria, 10,380 reports were included in this study. Among these, 1135 (10.9%) reports were related to pneumococcal vaccines, of which 668 (58.9%) were related to PPSV and 467 (41.1%) to PCV. 

Compared to all other vaccines, a higher proportion of pneumococcal vaccine reports were from people aged 65 and above (Table 1). The proportion of spontaneous reports was predominant, and most of them were from doctors and regional pharmacovigilance centers (RPVCs). According to subtype, a higher proportion of the PPSV reports involved the elderly compared to PCV reports (32.3% vs. 12.6%, respectively). The proportions of spontaneous reports were 98.0% and 56.1% for PPSVs and PCVs, respectively. 

We found 2262 AE-pairs for the pneumococcal vaccine, of which 37.8% were application site disorders (Table 2). The proportions of general disorders (24.9%), musculoskeletal system disorders (16.3%), skin and appendages disorders (6.3%), and central & peripheral nervous system disorders (6.0%) were higher for the pneumococcal vaccine than for all other vaccines. We found 1563 and 699 AE-pairs for PPSV and PCV, respectively, with application site disorders being predominant for both subtypes (36.4% in PPSV and 42.8% in PCV). However, there were differences in the proportions of AE-pairs between PPSV and PCV in musculoskeletal system disorders (19.5% and 9.4%, respectively), skin and appendages disorders (3.8% and 12.4%, respectively), central and peripheral nervous system disorders (7.6% and 2.4%, respectively), and respiratory system disorders (1.9% and 4.5%, respectively). We also observed a substantial difference between PPSV and PCV under skin and appendages disorders, and this difference was also noted in the subgroup analyses by age group. (18 years old or younger: PPSV 0.0%, PCV 11.2%; 19–64 years old: PPSV 8.7%, PCV 12.6%; 65 years old or older: PPSV 4.9%, PCV 12.6%) (Table S2).

### 3.2. Signal Detection

There was discrepancy in the number of signals generated for all pneumococcal vaccines by each algorithm (Table 2). IC generated the most signals (40 signals out of 101 AEs), while PRR, ROR, and tree-based scan statistic generated a similar number of signals, with PRR and ROR both generating 19 signals and tree-based scan statistic generating 15 signals; EBGM only generated 1 signal. Notably, cellulitis was the only AE detected by all algorithms. Results from subgroup analyses were generally inconsistent with those from the main analysis, with one additional signal (pharyngitis) detected for subgroup of age 19 years or younger; this AE term was known AE listed in the labeling information (Table S3).

Different results were obtained for the two vaccine subtypes using the three methods (Table 3). In the PPSV analysis, the tree-based scan statistic generated the highest number of signals (14), while IC generated 13 signals and the EBGM generated 7 signals. Moreover, 7 AEs were detected as signals using all three methods at the same time. In the case of PCVs, the disproportionality methods generated the highest number of signals (15), followed by tree-based scan statistic, which detected 6 signals and EBGM, which detected 3 signals. Only cellulitis was detected simultaneously from PPSVs and PCVs with all statistical methods.

### 3.3. Performance Evaluation

**Figure 2:**
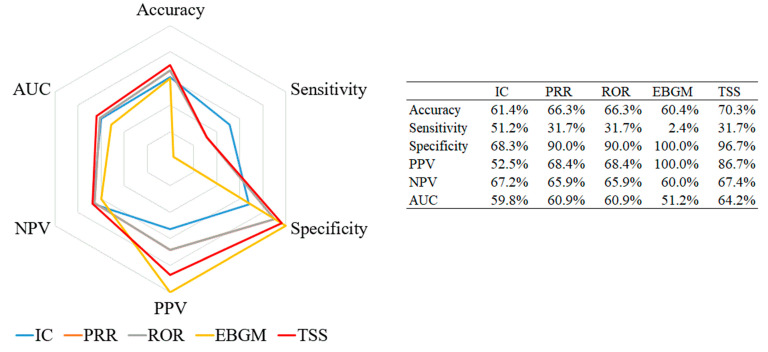
Radar chart of performance measurements for signal detection algorithms about pneumococcal vaccine. Abbreviations: IC, information component; PRR, proportional reporting ratio; ROR, reporting odds ratio; EBGM, empirical Bayes geometric mean; TSS, tree-based scan statistic; PPV, positive predicted value; NPV, negative predicted value; AUC, area under the curve.

All performance measurements were calculated with the pre-specified reference standard in terms of the WHO-ART PT level (Figure 2). The tree-based scan statistic showed the highest values for three measurements, namely accuracy (70.3%), NPV (67.4%), and AUC (64.2%), while EBGM showed 100% specificity and PPV. Sensitivity was the highest for IC (51.2%), whereas PRR and ROR did not show any measurements higher than those for the other algorithms.

## 4. Discussion

This study identified safety signals for the pneumococcal vaccines using several algorithms. Among the signal detection algorithms, both the tree-based scan statistic and the disproportionality methods generated a comparable number of signals, whereas EBGM generated the least number of signals. Moreover, there were no overall safety concerns associated with pneumococcal vaccines, and similar results were observed in the subgroup analysis of the age groups. However, we found a possible relationship between PCV and cellulitis in the subgroup analysis of pneumococcal vaccines, which warrants further clinical evaluation to confirm the causal relationship.

Although this is not the first study to implement tree-based scan statistic for safety surveillance, it is the first to test the applicability of tree-based scan statistic in the spontaneous reporting system. There are two major aspects to consider when using tree-based scan statistic, the first one being the construction of tree structured variable for analysis. Health claims databases use the International Classification of Diseases (ICD) codes to record medical diagnosis and related procedures [28], and it is often not suitable to fully capture drug-induced AEs. On the contrary, the spontaneous reporting system utilizes the WHO-ART and the Medical Dictionary for Regulatory Activities to record drug-induced AEs. Tree-based scan statistic has previously been applied in the health claims databases to screen unexpected AEs of pharmaceutical products, and one of its limitation was the use of ICD codes through Clinical Classification Software for defining the drug-induced AEs [29]. Our study tested the applicability of the tree-based scan statistic in the KAERS, where AEs are coded using the WHO-ART. As the WHO-ART is constructed as a hierarchical structure at pre-specified granularity, we believe that tree-based scan statistic is well-suited for screening unexpected AEs in the spontaneous reporting database.

The second aspect to consider when using tree-based scan statistic is the calculation of expected counts for the drug–AE pairs. In a previous study using health claims database [9], expected values were calculated using the number of events (as the numerator) and the follow-up time (as the denominator). In the case of spontaneous reports, due to a lack of follow-up time, two by two tables for each AE-pair are tabulated and then the total number of events, excluding the event of interest, is used as an alternative to the follow-up time. Such event counts replace the follow-up time, as expected values are routinely calculated using person-time or the number of people exposed in the health claims database while such information is not presented in the spontaneous database [23]. Even though under-reporting can be a limitation, the denominator used in our study was the most suitable to replace the follow-up time in the passive surveillance database. Additionally, the result of tree-based scan statistic was similar to that obtained using disproportionality methods, and the overall performance of tree-based scan statistic was higher than those for other algorithms. Therefore, we believe that the tree-based scan statistic has been applied successfully in the spontaneous reporting database.

Similar findings were observed compared to other studies [30,31]. EBGM was a more conservative method compared to the disproportionality methods as it generated the least number of signals for the pneumococcal vaccine. However, it should be noted that EBGM generated more signals when the signal detection analyses were conducted for PPSV and PCV. This may have resulted from differences in the contingency table according to each analysis unit. The EBGM is sensitive to the number of rows and columns because the expected values using the empirical Bayes method are calculated based on the size of the contingency table. Moreover, our primary result did not consider the difference in age distribution between the two subtypes of pneumococcal vaccine. This factor could have also diluted the result of the main analysis compared to the subgroup analysis.

With respect to the overall measurements, the EBGM showed extremely high specificity and PPV, but relatively low sensitivity. According to a previous study [32], the EBGM showed low sensitivity and high specificity and PPV when using a high threshold. Our results partly support these previous findings, although the sensitivity observed in our study was even lower-than-expected. This algorithm was developed for a large contingency table having millions of cells, and an example of the contingency table in an empirical study had approximately 1.3 million cells (with 4.9 million counts) [33]. Our database had about 4400 cells with 10,000 counts. The volume of the database may not have been enough for the EBGM to show a stable performance for signal detection compared to the reference standard. Nonetheless, the performance of EBGM is acceptable given its high specificity and PPV. This provides an efficient feature for routine post-marketing pharmacovigilance activity [34].

Our results are consistent with those of several studies on AEs following pneumococcal vaccine conducted in different countries. Post-licensure studies using the US Vaccine Adverse Event Reporting System showed that application site disorders including erythema, pain, and swelling were predominant in all age groups vaccinated with PPSV and PCV [35,36]. Additionally, AEs reported from phase II clinical safety trial in China were mostly AEs at the local injection site and non-serious systemic reactions such as myalgia and joint pain [37].

Moreover, cellulitis was detected as a safety signal for both PPSV and PCV, of which cellulitis is documented as a known AE in the reference standard for PPSV, but not in the reference standard for PCV. Whether cellulitis has previously been associated only with PPSV remains unclear. A few case reports and case series have introduced suspected cases of systemic inflammatory reactions representing the clinical presentation of cellulitis [38,39], whereas only a single study raised a potential association between PCV and cellulitis through a 12-year retrospective medical chart review. According to a previous study on the impact of the publicly funded PCV immunization program on the number of hospitalized cases of orbital cellulitis, a bacterial infection of the post-septal tissues of the eye, there was an increasing trend from 0.39 cases to 0.90 cases per 1000 admissions before and after introduction of the immunization program, respectively [40]. Cellulitis is an acute pyogenic inflammation of the dermis and subcutaneous tissue accompanied by tenderness, warmth, and swelling at the site of infection [41]. Given that the major causative bacteria of cellulitis are streptococci, it may be feasible to suspect the cellulitis reports as a disseminated infection from PCV. However, we assert this to be highly unlikely as PCV includes a non-virulent capsular polysaccharide of *Streptococcus pneumoniae*.

Results from both main and subgroup analyses show differential proportion of AEs of skin and appendage disorders between PPSV and PCV. Such difference may have been due to the target population eligible for pneumococcal vaccination under NIP, as PCV is routinely administered in children and adolescents and PPSV is administered in the elderly. The pediatric population generally experiences AEs related to the skin more frequently and widely than the adult population [42], and indeed, in the US, the most common AEs in children are general disorders and administration site conditions [43]. Therefore, the high proportion of skin-related AEs due to PCVs are understandable when considering the age distribution.

Early detection of safety signals using a spontaneous reporting database have been receiving growing attention given the importance of post-marketing safety surveillance of the pharmaceutical products including vaccines. While traditional pharmacoepidemiological methods have traditionally been used to identify and quantify AEs, they are time-consuming, require large databases, and most importantly, need a priori hypothesis. Signal detection algorithms used in this study have been utilized numerously to generate specific hypotheses for specific drug–event pairs, of which the tree-based scan statistics is a relatively novel data mining method that has rarely been implemented in the spontaneous reporting database. Their application in the spontaneous reporting database is practical and enables for early detection of potential safety issues which would be the first of many steps in reducing public health burden relating to the AEs following vaccination. Our study has several strengths. First, we used a nationwide spontaneous reporting database for all vaccines from 1988 to 2017. Second, diverse algorithms for signal detection were applied in this study to consider different spectrums according to different statistical approaches. Third, this study not only detected the safety signals, but also evaluated the performance of the algorithms to identify which is more suitable to conduct a post-marketing safety surveillance of pneumococcal vaccines. Finally, we identified the safety signal of a possible risk of cellulitis following PCV vaccination.

However, there are some limitations to this study. First, there is an inherent limitation of under-reporting in a passive surveillance system [44]. Therefore, our results should be interpreted with caution due to potential selection bias or under-estimation. Second, using the reference standard, which includes already known AEs of pneumococcal vaccines, could be an issue as the quantitative performance of each algorithm could be over- or under-estimated according to the reference standard [45]. However, we believe that the labeling information included all relevant AEs, as pneumococcal vaccines have been used for a long period since their approval [46,47]. Furthermore, the objective of our study was to identify a possible causal relationship between a vaccine and an AE [48]. Therefore, further pharmacoepidemiologic studies are needed to evaluate the association between cellulitis and PCV.

## 5. Conclusions

Overall, we identified a discrepancy in the results of signal detection observed among the three algorithms. Compared to the disproportionality methods, the EBGM generated the lowest number of signals, and the tree-based scan statistic generated either a lower or an equal number of signals. Additionally, both methods seemed to calibrate noise. In terms of performance, the tree-based scan statistic showed balanced measurements. Moreover, a possible causal relationship between PCVs and cellulitis was observed. Nonetheless, the findings should be interpreted with caution due to a lack of a gold standard for an algorithm for signal detection. Further pharmacoepidemiologic studies are therefore required to confirm the findings of this study.

## Figures and Tables

**Table 1:** Characteristics of adverse event reports related to pneumococcal vaccine and all other vaccines from 1988 to 2017.

Characteristics	Pneumococcal Vaccine						All Other Vaccines	
	ALL		PPSV		PCV			
	(N = 1135)		(N = 668)		(N = 467)		(N = 9245)	
	Reports %		Reports %		Reports %		Reports %	
**Sex**								
Female	674	(59.4)	389	(58.2)	285	(61.0)	6346	(68.6)
Male	455	(40.1)	274	(41.0)	181	(38.8)	2824	(30.5)
Missing	6	(0.5)	5	(0.7)	1	(0.2)	75	(0.8)
**Age group (years)**								
<2	98	(8.6)	0	(0.0)	98	(21.0)	1252	(13.5)
2–11	16	(1.4)	3	(0.4)	13	(2.8)	531	(5.7)
12–18	3	(0.3)	3	(0.4)	0	(0.0)	220	(2.4)
19–64	313	(27.6)	54	(8.1)	259	(55.5)	4246	(45.9)
65	275	(24.2)	216	(32.3)	59	(12.6)	260	(2.8)
Missing	430	(37.9)	392	(58.7)	38	(8.1)	2736	(29.6)
**Report type**								
Spontaneous report	918	(80.9)	656	(98.2)	262	(56.1)	5700	(61.7)
Research	190	(16.7)	0	(0.0)	190	(40.7)	3460	(37.4)
Other	27	(2.4)	12	(1.8)	15	(3.2)	85	(0.9)
**Report source by professions**								
Doctor	724	(63.8)	428	(64.1)	296	(63.4)	3654	(39.5)
Pharmacist	29	(2.6)	5	(0.7)	24	(5.1)	2221	(24.0)
Nurse	107	(9.4)	30	(4.5)	77	(16.5)	592	(6.4)
Consumer	13	(1.1)	3	(0.4)	10	(2.1)	848	(9.2)
Other *	226	(19.9)	183	(27.4)	43	(9.2)	1536	(16.6)
Missing	36	(3.2)	19	(2.8)	17	(3.6)	394	(4.3)
**Report source by affiliation**								
RPVC	677	(59.6)	452	(67.7)	225	(48.2)	4450	(48.1)
Pharmaceutical company	218	(19.2)	21	(3.1)	197	(42.2)	3742	(40.5)
Medical institution	60	(5.3)	49	(7.3)	11	(2.4)	52	(0.6)
Customer ^†^	33	(2.9)	25	(3.7)	8	(1.7)	153	(1.7)
Other	147	(13.0)	121	(18.1)	26	(5.6)	848	(9.2)

Abbreviations: PPSV, pneumococcal polysaccharide vaccine; PCV, pneumococcal conjugate vaccine; AE, adverse event; RPVC, regional pharmacovigilance center. * Other included lawyer and other healthcare professionals. ^†^ Other included pharmacy and health center.

**Table 2:** The frequency of adverse events for pneumococcal vaccine and all other vaccines from 1988 to 2017.

Adverse Event	Pneumococcal Vaccine						All Other Vaccines	
(WHO-ART System-Organ Class)	All		PPSV		PCV			
	(N = 2262)		(N = 1563)		(N = 699)		(N = 16295)	
	AE-Pairs	%	AE-Pairs	%	AE-Pairs	%	AE-Pairs	%
Application site disorders	855	(37.8)	569	(36.4)	286	(42.8)	6845	(42.0)
Body as a whole - general disorders	564	(24.9)	384	(24.6)	180	(26.9)	3589	(22.0)
Musculoskeletal system disorders	368	(16.3)	305	(19.5)	63	(9.4)	1669	(10.2)
Skin and appendages disorders	142	(6.3)	59	(3.8)	83	(12.4)	763	(4.7)
Central & peripheral nervous system disorders	135	(6.0)	119	(7.6)	16	(2.4)	833	(5.1)
Gastro-intestinal system disorders	69	(3.0)	51	(3.3)	18	(2.7)	585	(3.6)
Respiratory system disorders	60	(2.7)	30	(1.9)	30	(4.5)	724	(4.4)
Psychiatric disorders	21	(0.9)	12	(0.8)	9	(1.3)	534	(3.3)
Metabolic and nutritional disorders	12	(0.5)	11	(0.7)	1	(0.1)	184	(1.1)
Resistance mechanism disorders	7	(0.3)	1	(0.1)	6	(0.9)	39	(0.2)
Cardiovascular disorders, general	6	(0.3)	6	(0.4)	0	(0.0)	23	(0.1)
Heart rate and rhythm disorders	5	(0.2)	5	(0.3)	0	(0.0)	10	(0.1)
Vascular (extracardiac) disorders	5	(0.2)	3	(0.2)	2	(0.3)	4	(0.0)
Vision disorders	3	(0.1)	2	(0.1)	1	(0.1)	15	(0.1)
Urinary system disorders	3	(0.1)	2	(0.1)	1	(0.1)	12	(0.1)
Hearing and vestibular disorders	2	(0.1)	2	(0.1)	0	(0.0)	6	(0.0)
Liver and biliary system disorders	2	(0.1)	0	(0.0)	2	(0.3)	12	(0.1)
White cell and RES* disorders	1	(0.0)	1	(0.1)	0	(0.0)	400	(2.5)
Platelet, bleeding & clotting disorders	1	(0.0)	1	(0.1)	0	(0.0)	14	(0.1)
Reproductive disorders, female	1	(0.0)	0	(0.0)	1	(0.1)	6	(0.0)

Abbreviations: WHO-ART, World Health Organization Adverse Reactions Terminology; PPSV, pneumococcal polysaccharide vaccine; PCV, pneumococcal conjugate vaccine; AE, adverse event; RES, reticuloendothelial system.

**Table 3:** Signal detection of pneumococcal vaccine using the disproportionality methods, empirical Bayes geometric mean, and tree-based scan statistic from 1988 to 2017.

Adverse Event * (WHO-ART PT Level)	No. of AE-Pairs	IC	PRR	ROR	EBGM	*p*-Value for TSS	Listed in Labeling ^†^	Signal Detection				
								IC ^‡^	PRR ^§^	ROR ^‖^	EBGM	TSS ^#^
**Pneumococcal vaccine (All)**												
Myalgia	314	0.35	1.54	1.62	1.47	0.0010	O	O				O
Fever	304	0.66	2.03	2.19	1.94	0.0010	O	O	O	O		O
Injection site reaction	201	0.12	1.32	1.35	1.28	0.0010	O	O				O
Injection site discharge	195	0.66	2.09	2.19	1.84	0.0010	O	O	O	O		O
Rigors	108	0.42	1.81	1.85	1.55	0.0010	O	O				O
Dizziness	57	0.34	1.83	1.85	1.49	0.0010		O				O
Rash	52	0.10	1.52	1.53	1.29	0.0400	O	O				O
Arthralgia	45	0.46	2.13	2.16	1.59	0.0020	O	O	O	O		O
Cellulitis	43	2.06	22.13	22.54	4.59	0.0010	O	O	O	O	O	O
Urticaria	31	0.29	1.98	1.99	1.47	0.0060	O	O				O
Asthenia	23	-0.15	1.43	1.43	1.03	0.0030	O	O				O
Oedema	19	0.82	3.91	3.94	1.82	0.0030	O	O	O	O		O
Injection site inflammation	13	0.76	4.26	4.28	1.71	0.0100		O	O	O		O
Dyspnoea	13	0.62	3.60	3.62	1.58	0.0260	O	O	O	O		O
Angioedema	12	1.10	7.20	7.24	1.91	0.0040	O	O	O	O		O
**Pneumococcal polysaccharide vaccine**												
Myalgia	256	0.57	1.82	1.98	2.00	0.0010	O	O			O	O
Fever	163	0.25	1.45	1.51	1.70	0.0010	O	O				O
Injection site discharge	161	0.89	2.48	2.65	2.52	0.0010	O	O	O	O	O	O
Rigors	99	0.82	2.45	2.54	2.34	0.0010	O	O	O	O	O	O
Headache	54	-0.03	1.32	1.33	1.32	0.0040	O	O				O
Dizziness	53	0.75	2.53	2.58	2.25	0.0010		O	O	O	O	O
Arthralgia	43	0.91	3.04	3.09	2.43	0.0010	O	O	O	O	O	O
Nausea	26	0.19	1.79	1.80	1.49	0.0090	O	O				O
Urticaria	21	0.19	1.86	1.87	1.49	0.0050	O	O				O
Cellulitis	19	1.28	5.44	5.49	2.64	0.0010	O	O	O	O	O	O
Asthenia	19	0.07	1.72	1.73	1.31	0.0010	O	O				O
Angioedema	10	1.34	7.77	7.81	2.02	0.0020	O	O	O	O	O	O
**Pneumococcal conjugate vaccine**												
Fever	141	1.17	2.90	3.38	2.06	0.0010	O	O	O	O	O	O
Injection site reaction	102	0.76	2.17	2.37	1.49	0.0010	O	O	O	O		O
Rash	40	1.37	3.95	4.12	2.18	0.0010	O	O	O	O	O	O
Cellulitis	24	2.82	18.58	19.21	4.32	0.0010		O	O	O	O	O
Pruritus	15	0.77	2.95	2.99	1.32	0.1150		O	O	O		O
Pharyngitis	13	0.24	2.01	2.03	0.91	0.0190		O	O	O		O
Oedema	11	1.61	6.54	6.62	1.77	0.0040	O	O	O	O		O
Rash erythematous	10	1.02	3.99	4.04	1.30	0.1230		O	O	O		
Coughing	10	0.57	2.75	2.77	1.04	0.6370		O	O	O		

Abbreviations: WHO-ART, World Health Organization-Adverse Reactions Terminology; PT, preferred term; AE, adverse events; IC, information component; PRR, proportional reporting ratio; ROR, reporting odds ratio; EBGM, empirical Bayes geometric mean; TSS, tree-based scan statistic. ^*^ Table showed only the adverse events satisfying two conditions as follow: (1) more than 10 frequencies; (2) detected by at least one algorithm. ^†^ Adverse events were checked where these were listed in the labeling information either in the Food and Drug Administration of United States or the Ministry of Food and Drug Safety of Korea. ^‡^ Safety signals using IC were defined as adverse events where the lower bound of the 95% confidence intervals was greater than zero. ^§^ Safety signals using PRR were defined as adverse events where thresholds of PRR was greater than two. ^‖^ Safety signals using ROR were defined as adverse events where thresholds of ROR was greater than two. ^¶^ Safety signals using tree-based scan statistic were defined as adverse events detected at a 0.05 level of significance. ^#^ Safety signals using EBGM were defined as adverse events where the lower bound of the 90% confidence intervals was greater than or equal to two.

## Supplementary Materials

The following are available online at https://www.mdpi.com/2076-393X/8/2/242/s1, Figure S1: Detailed formula used for the performance evaluation using a confusion matrix, Table S1: List of vaccines in Korea Institute of Drug Safety & Risk Management-Korea Adverse Event Reporting System Database, Table S2: Subgroup analysis for the frequency of adverse events for pneumococcal vaccine and all other vaccines from 1988 to 2017, Table S3: Subgroup analysis for signal detection of pneumococcal vaccine using the disproportionality methods, empirical Bayes geometric mean, and tree-based scan statistic from 1988 to 2017.
