# Supplementary material for: Safety Surveillance of Pneumococcal Vaccine Using Three Algorithms: Disproportionality Methods, Empirical Bayes Geometric Mean, and Tree-Based Scan Statistic
Source: Vaccines (Basel). 2020 May 22;8(2):242. doi: 10.3390/vaccines8020242 (PMC7349998; doi:10.3390/vaccines8020242)
Supplement: Supplementary file 1 [file vaccines-08-00242-s001.pdf]

Supplementary

Table S1. List of vaccines in Korea Institute of Drug Safety & Risk Management-Korea Adverse Event Reporting System Database.

Vaccine	Subtype
BCG	BCG Danish strain
	BCG Tokyo strain
Diphtheria, Tetanus, Pertussis	DTaP
	DTwP
	Tdap
	Td
	DTaP-IPV
	DTaP-IPV/Hib
Hepatitis A	Hepatitis A, inactivated
Hepatitis B	Hepatitis B, recombinant
Haemophilus influenzae B	Haemophilus influenzae, conjugated
Human Papilloma virus	HPV2 (types 16, 18)
	HPV4 (types 6, 11, 16, 18)
	HPV9 (types 6, 11, 16, 18, 31, 33, 45, 52, 58)
Herpes Zoster	Herpes zoster virus, live attenuated
Influenza	Influenza, inactivated
	Influenza, live attenuated
Japanese encephalitis	Japanese encephalitis, inactivated
	Japanese encephalitis, live attenuated
Meningococcal	Meningococcal (serogroups A, C, W, Y)
	Meningococcal (serogroup B)
MMR	Measles, Mumps, Rubella, live attenuated
Polio	Polio virus, inactivated
	Polio virus, oral, live attenuated
Pneumococcal	Pneumococcal conjugate vaccine 13
	Pneumococcal polysaccharide vaccine 23
Typhoid	Typhoid conjugate, oral
	Vi capsular polysaccharide
Rota virus	Rota virus, live attenuated
Varicella	Varicella zoster virus, live attenuated
Yellow fever	Yellow fever, live attenuated

Abbreviations: BCG, Bacille Calmette-Guérin; DTaP, Diphtheria, tetanus, acellular pertussis; DTwP, Diphtheria, tetanus, whole cell pertussis; Tdap, Tetanus, reduced diphtheria, acellular pertussis; Td, Tetanus, diphtheria toxoid; IPV, Inactivated polio virus; Hib, Haemophilus influenza; HPV, Human papilloma virus.

Table S2. Subgroup analysis for the frequency of adverse events for pneumococcal vaccine and all other vaccines from 1988 to 2017.

**Adverse Event ***	**Pneumococcal Vaccine**						**All Other Vaccines**	
**(WHO-ART System-Organ Class)**	**All**		**PPSV**		**PCV**			
	**AE-Pairs**	**%**	**AE-Pairs**	**%**	**AE-Pairs**	**%**	**AE-Pairs**	**%**
**18 years old or younger (N = 3516)**								
Total	142	(100.0)	8	(100.0)	134	(100.0)	3374	(100.0)
Body as a whole - general disorders	68	(47.9)	3	(37.5)	65	(48.5)	829	(24.6)
Respiratory system disorders	19	(13.4)	0	(0.0)	19	(14.2)	78	(2.3)
Application site disorders	18	(12.7)	4	(50.0)	14	(10.4)	974	(28.9)
Skin and appendages disorders	15	(10.6)	0	(0.0)	15	(11.2)	209	(6.2)
**19-64 years old (N = 8910)**								
Total	530	(100.0)	103	(100.0)	427	(100.0)	8380	(100.0)
Application site disorders	261	(49.2)	37	(35.9)	224	(52.5)	3587	(42.8)
Body as a whole - general disorders	100	(18.9)	26	(25.2)	74	(17.3)	1898	(22.6)
Skin and appendages disorders	63	(11.9)	9	(8.7)	54	(12.6)	381	(4.5)
Musculoskeletal system disorders	56	(10.6)	10	(9.7)	46	(10.8)	1192	(14.2)
Central & peripheral nervous system disorders	16	(3.0)	6	(5.8)	10	(2.3)	496	(5.9)
Gastro-intestinal system disorders	14	(2.6)	6	(5.8)	8	(1.9)	178	(2.1)
Respiratory system disorders	12	(2.3)	5	(4.9)	7	(1.6)	421	(5.0)
**65 years old or older (N = 918)**								
Total	457	(100.0)	370	(100.0)	87	(100.0)	461	(100.0)
Application site disorders	181	(39.6)	150	(40.5)	31	(35.6)	94	(20.4)
Body as a whole - general disorders	127	(27.8)	109	(29.5)	18	(20.7)	121	(26.2)
Musculoskeletal system disorders	50	(10.9)	35	(9.5)	15	(17.2)	75	(16.3)
Skin and appendages disorders	29	(6.3)	18	(4.9)	11	(12.6)	59	(12.8)
Central & peripheral nervous system disorders	28	(6.1)	23	(6.2)	5	(5.7)	44	(9.5)
Gastro-intestinal system disorders	15	(3.3)	12	(3.2)	3	(3.4)	17	(3.7)
Respiratory system disorders	12	(2.6)	9	(2.4)	3	(3.4)	33	(7.2)

Abbreviations: WHO-ART, World Health Organization Adverse Reactions Terminology; PPSV, pneumococcal polysaccharide vaccine; PCV, pneumococcal conjugate vaccine; AE, adverse event; RES, reticuloendothelial system. * Table showed only the adverse events more than 10 frequencies in pneumococcal vaccine.

Table S3. Subgroup analysis for signal detection of pneumococcal vaccine using the disproportionality methods, empirical Bayes geometric mean, and tree-based scan statistic from 1988 to 2017.

Adverse Event * (WHO-ART PT Level)	No. of AE-Pairs	IC	PRR	ROR	EBGM	*p*-Value for TSS	Listed in Labeling †	Signal Detection IC ‡	PRR §	ROR ‖	EBGM ¶	TSS #
**18 years old or younger**												
Fever	57	1.22	3.50	5.18	2.06	0.001	O	O	O	O	O	O
Pharyngitis	12	2.02	10.18	11.03	2.12	0.001	O	O	O	O	O	O
**19–64 years old**												
Injection site reaction	93	1.06	2.92	3.33	1.88	0.001	O	O	O	O		O
Fever	65	1.09	3.12	3.42	1.91	0.001	O	O	O	O		O
Injection site discharge	29	0.37	1.98	2.03	1.13	0.430	O	O		O		
Rash	27	0.99	3.34	3.46	1.43	0.002	O	O	O	O		O
Cellulitis	23	3.08	121.22	126.67	5.01	0.001	O	O	O	O	O	O
Pruritus	14	0.85	3.46	3.53	1.43	0.047		O	O	O		O
**65 years old or older**												
Fever	93	0.20	2.93	3.43	1.20	0.024	O	O	O	O		O
Injection site reaction	79	0.18	3.07	3.50	1.19	0.019	O	O	O	O		O
Injection site discharge	25	-0.01	5.04	5.28	1.10	0.320	O		O	O		
Cellulitis	14	-0.19	7.06	7.25	0.99	0.829	O		O	O		

Abbreviations: WHO-ART, World Health Organization-Adverse Reactions Terminology; PT, preferred term; AE, adverse events; IC, information component; PRR, proportional reporting ratio; ROR, reporting odds ratio; EBGM, empirical Bayes geometric mean; TSS, tree-based scan statistic. * Table shows only the adverse events satisfying two conditions as follows: 1) more than 10 frequencies; 2) detected by at least one algorithm. † Adverse events were checked where these were listed in the labeling information either in the Food and Drug Administration of United States or the Ministry of Food and Drug Safety of Korea. ‡ Safety signals using IC were defined as adverse events where the lower bound of the 95% confidence intervals was greater than zero. § Safety signals using PRR were defined as adverse events where thresholds of PRR was greater than two. ‖ Safety signals using ROR were defined as adverse events where thresholds of ROR was greater than two. ¶ Safety signals using tree-based scan statistic were defined as adverse events detected at a 0.05 level of significance. # Safety signals using EBGM were defined as adverse events where the lower bound of the 90% confidence intervals was greater than or equal to two.

	Adverse event in labeling	Adverse event not in labeling
Adverse events defined as signals	a (True positive)	b (False positive)
Adverse events did not defined as signals	c (False negative)	d (True negative)

– Accuracy = (a+d) / (a+b+c+d)
– Sensitivity = a / (a+c)
– Specificity = d / (b+d)
– Positive Predictive Value = a / (a+b)
– Negative Predictive Value = d / (c+d)
– Area Under the Curve = 1/2 × (Sensitivity+Specificity)

Figure S1. Detailed formula used for the performance evaluation using a confusion matrix.
